# Two ferric uptake regulator proteins in *Ralstonia pseudosolanacearum* strain OE1-1 function cooperatively in response to the extracellular iron level under ferrous iron-rich conditions

**DOI:** 10.1128/aem.02276-25

**Published:** 2026-03-02

**Authors:** Sora Tateda, Yuki Terazawa, Akinori Kiba, Kouhei Ohnishi, Yasufumi Hikichi, Masayuki Tsuzuki

**Affiliations:** 1 Faculty of Agriculture and Marine Science, Kochi University 74261 , Nankoku, Kochi, Japan; The University of Arizona, Tucson, Arizona, USA

**Keywords:** transcriptional regulator, iron, siderophore, *Ralstonia solanacearum* species complex, phytopathogenic bacteria

## Abstract

Soil-borne Gram-negative bacteria in the *Ralstonia solanacearum* species complex (RSSC) cause bacterial wilt symptoms in diverse crop plants. Although iron is an important metal ion for most organisms, the regulatory effect of iron on soil-borne RSSC gene expression remains unknown. Thus, we identified two ferric uptake regulator (Fur) proteins, Fur1 and Fur2, in *R. pseudosolanacearum* strain OE1-1. All four RSSC phylotype strains had two Fur homologs, a conserved Fur (Fur1) and an alternative Fur (Fur2). We also analyzed the transcriptomes of a *fur1* deletion mutant, *fur2* deletion mutant, and *fur1*⁄*fur2* double deletion mutant, revealing that Fur1 and Fur2 cooperatively repress siderophore-related gene expression under Fe^2+^-rich conditions. Furthermore, extracellular Fe^3+^-chelating activity was cooperatively controlled by Fur1 and Fur2 under Fe^2+^-rich conditions. Additionally, nitrate metabolism-related gene expression levels were upregulated only in the *fur1*⁄*fur2* double deletion mutant under Fe^2+^-rich conditions. This double deletion mutant had a lower growth level than either single deletion mutant. Virulence assays involving tomato plants revealed that single deletions (*fur1 *or *fur2*) moderately decreased virulence, whereas the double deletion (*fur1* and *fur2*) resulted in a significant decrease in virulence. Considered together, these results suggest that the two Fur homologs cooperatively regulate gene expression under Fe^2+^-rich conditions to control strain OE1-1 iron uptake, growth, and virulence.

**IMPORTANCE:**

The *Ralstonia solanacearum* species complex (RSSC) comprising soil-borne Gram-negative phytopathogenic bacteria causes bacterial wilt diseases of diverse crop plants. Considering that phylotype I strain OE1-1 enters iron-rich roots from iron-deficient soil during an infection of tomato plants, the mechanisms controlling strain OE1-1 gene expression in response to extracellular iron levels should be clarified. In this study, RSSC was revealed to have two ferric uptake regulator homologs (Fur1 and Fur2). Notably, Fur1 and Fur2 cooperatively repress the expression of genes related to siderophores (Fe^3+^-chelating compounds) as well as the extracellular Fe^3+^-chelating activity in the presence of sufficient amounts of extracellular Fe^2+^. Additionally, Fur1 and Fur2 contribute to the virulence of strain OE1-1 in tomato plants. These findings suggest that RSSC uses two Fur proteins to modulate extracellular Fe^3+^-chelating activities in response to extracellular iron levels to maintain virulence in crop plants.

## INTRODUCTION

The soil-borne, Gram-negative β-proteobacterium *Ralstonia solanacearum* species complex (RSSC) causes bacterial wilt in a wide range of host plant species, resulting in devastating crop losses worldwide (1). RSSC in soil infects host plants through the roots and then moves to vascular tissues, wherein bacteria proliferate extensively (2–5). RSSC reportedly responds to the substantial environmental changes occurring during the transition from nutrient-limited soil to nutrient-rich host plants (6). Elucidating how RSSC adapts and thrives under various environmental conditions has important implications for pathogen control.

Iron uptake is an important process in various organisms, including bacteria, because some enzymes require iron as a co-factor during redox reactions as well as proteins that bind to iron via metal-binding domains (7). Acquisition of abundant iron from hosts can be a reason why some bacteria infect other organisms (7). On the other hand, excessive amounts of intracellular iron may be harmful to bacteria because free Fe^2+^ leads to the production of hydroxyl radicals (8). Thus, bacterial cells must be able to sense extracellular iron levels and control intracellular iron levels. Iron has two different redox states with different characteristics: ferrous iron (Fe^2+^) and ferric iron (Fe^3+^). It has been estimated that iron redox state in the cytoplasm favors Fe^2+^ because of the solubility in the cytoplasm, and Fe^2+^ is generally the form incorporated into iron-dependent enzymes (9, 10). However, Fe^3+^, which is relatively unreactive and insoluble, is abundant under natural conditions. Also, in hosts, abundant iron is likely to be pooled in various ways as the labile iron pool, which requires bacteria to have specific mechanisms to obtain iron from hosts (7, 10). To take up environmental and/or host-derived Fe^3+^, bacterial cells produce siderophores, which are Fe^3+^-chelating secondary metabolites (11). In many bacterial species, siderophore production is controlled by a conserved transcriptional regulator family, ferric uptake regulator (Fur), whose members contain a DNA-binding domain and metal-binding motifs (12). The binding of Fe^2+^ to the metal-binding motif of Fur proteins alters the DNA-binding domain, thereby enabling Fur to bind to specific DNA motifs in the presence of Fe^2+^. In some bacterial species, the expression of siderophore-related genes is regulated by Fur proteins.

RSSC strains reportedly produce two siderophores: micacocidin (13) and staphyloferrin B (14). Micacocidin has a yersiniabactin-like structure and is biosynthesized by enzymes encoded at the *mic* locus in the RSSC genome (13). Staphyloferrin B is a polycarboxylate siderophore produced by enzymes encoded in the gene cluster that includes *ssd*, whose expression level increases under iron-deficient conditions (14). However, whether Fur proteins regulate siderophore production in RSSC remains unknown.

To elucidate the mechanism regulating RSSC gene expression in response to extracellular iron levels, we functionally characterized Fur proteins in *R. pseudosolanacearum* strain OE1-1. We determined that RSSC has an alternative Fur clade protein (Fur2) in addition to a conserved Fur (Fur1). A transcriptome analysis was performed to analyze the effects of Fur1 and Fur2 on gene expression. Moreover, we examined the effects of Fur1 and Fur2 on the Fe^3+^-chelating activity and growth of *R. pseudosolanacearum* strain OE1-1, while also investigating whether Fur1 and Fur2 contribute to the virulence of strain OE1-1.

## RESULTS

### The RSSC genome contains two genes encoding a conserved Fur and an additional non-conserved Fur exclusive to RSSC

To identify Fur proteins in RSSC, we first searched the *R. pseudosolanacearum* strain OE1-1 genome and identified two genes, *fur1* (*RSc2747*) and *fur2* (*RSp0247*), which encode Fur1 and Fur2, respectively. We obtained two putative Fur protein sequences from representative strains of four RSSC phylotypes. These sequences were aligned to compare domain structures. Fur1 and Fur2 in RSSC retained the N-terminal DNA-binding domain and two metal-binding sites conserved in the Fur proteins of the distantly related species *Escherichia coli* and *Helicobacter pylori* (15, 16), suggesting that Fur1 and Fur2 are Fur family members that bind to DNA and metal ions (Fig. S1).

**Fig 1:**
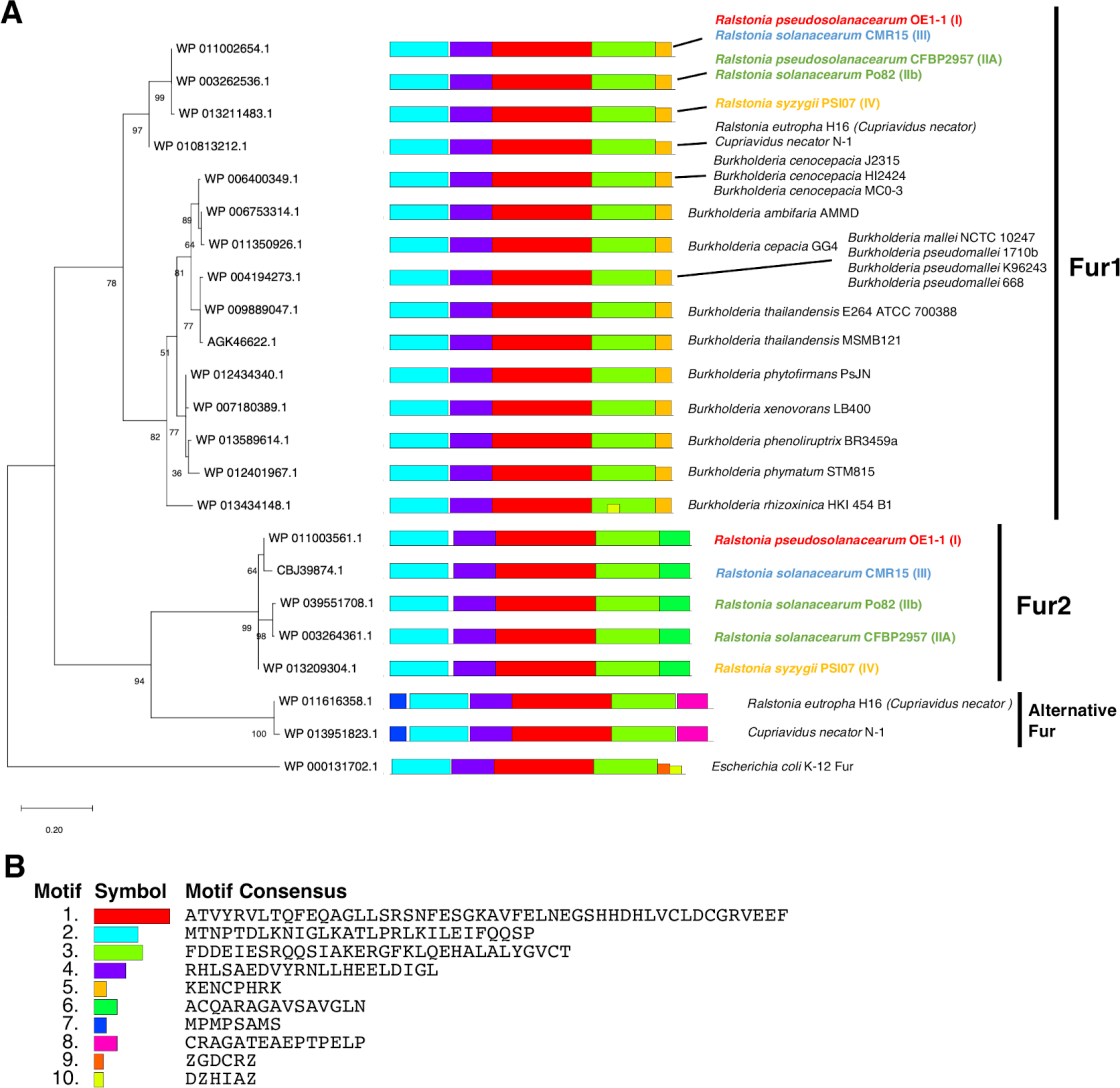
*Ralstonia solanacearum* species complex has two ferric uptake regulator homologous proteins in different clades. (**A**) A phylogenetic tree and putative amino acid motifs predicted by a MEME analysis revealed relationships between putative ferric uptake regulator (Fur) family proteins in *Ralstonia solanacearum* species complex (RSSC) and other closely related non-RSSC *Burkholderiaceae* species. The numbers written at each branch are those of the percentage of the result of a total of 1,000 times bootstrapping, supporting the branch. Protein IDs are provided along with strain name(s). Some protein IDs are shared by multiple species because the amino acid sequences were identical. For RSSC species, phylotype numbers are provided and distinguished by color: red: phylotype I, green: phylotypes IIA and IIB; blue: phylotype III; and yellow: phylotype IV. The Fur amino acid sequence in *Escherichia coli* strain K-12 (WP_000131702.1) was used as an outgroup. Different conserved motifs predicted by a MEME analysis are distinguished by differentially colored boxes. The sequences having the same amino acid motif are grouped as Fur1 or Fur2. (**B**) Motif IDs and motif consensus amino acid sequences shown in panel A, as provided by a MEME analysis, are displayed.

Considering RSSC belongs to the family *Burkholderiaceae* (β-proteobacteria) (17), we obtained 22 putative Fur sequences from non-RSSC *Burkholderiaceae* species and one from *E. coli* as an outgroup for a phylogenetic analysis. In the resulting phylogenetic tree, Fur1 and Fur2 were clustered in different groups (Fig. 1A). Only four RSSC phylotypes and two strains of the closest species (*Cupriavidus necator*, which is also known as *Ralstonia eutropha*) had proteins in the additional clade containing *R. pseudosolanacearum* Fur2. By contrast, the other *Burkholderiaceae* species only had proteins belonging to the clade containing *R. pseudosolanacearum* Fur1 (Fig. 1A).

A MEME analysis conducted to compare domain structures identified 10 conserved amino acid motifs and revealed that Fur1 in RSSC and Fur in *Burkholderia* species have the same motif structures, suggesting that RSSC Fur1 and other *Burkholderia* Fur proteins have conserved functions, which is grouped as Fur1 (Fig. 1A and B). Fur2 in RSSC and two Fur proteins in *C. necator* (WP_01161616358.1 and WP_0135951823.1) differed in terms of their C-terminal motif(s) and additional N-terminal motif #7 in *C. necator* Fur proteins, suggesting that they are grouped into different clades: Fur2 and alternative Fur (Fig. 1A and B). The protein structures of Fur1 and Fur2 were predicted by AlphaFold 3, and it was suggested that C-terminal amino acids in Fur1 do not form a static structure, whereas those in Fur2 form α-helix (Fig. S2). The phylogenetic analysis and the MEME analysis combining AlphaFold analysis suggested that Fur1 and Fur2 are tentatively in a paralogous relationship with different functional characters, whose ancestor-encoding genes underwent the duplication before the divergence of RSSC phylotypes. These results suggest that the presence of an alternative Fur2 clade protein in addition to the conserved Fur1 protein is a feature of RSSC species (Fig. 1A and B).

### Fur1 and Fur2 control siderophore-related gene expression under Fe^2+^-rich conditions

**Fig 2:**
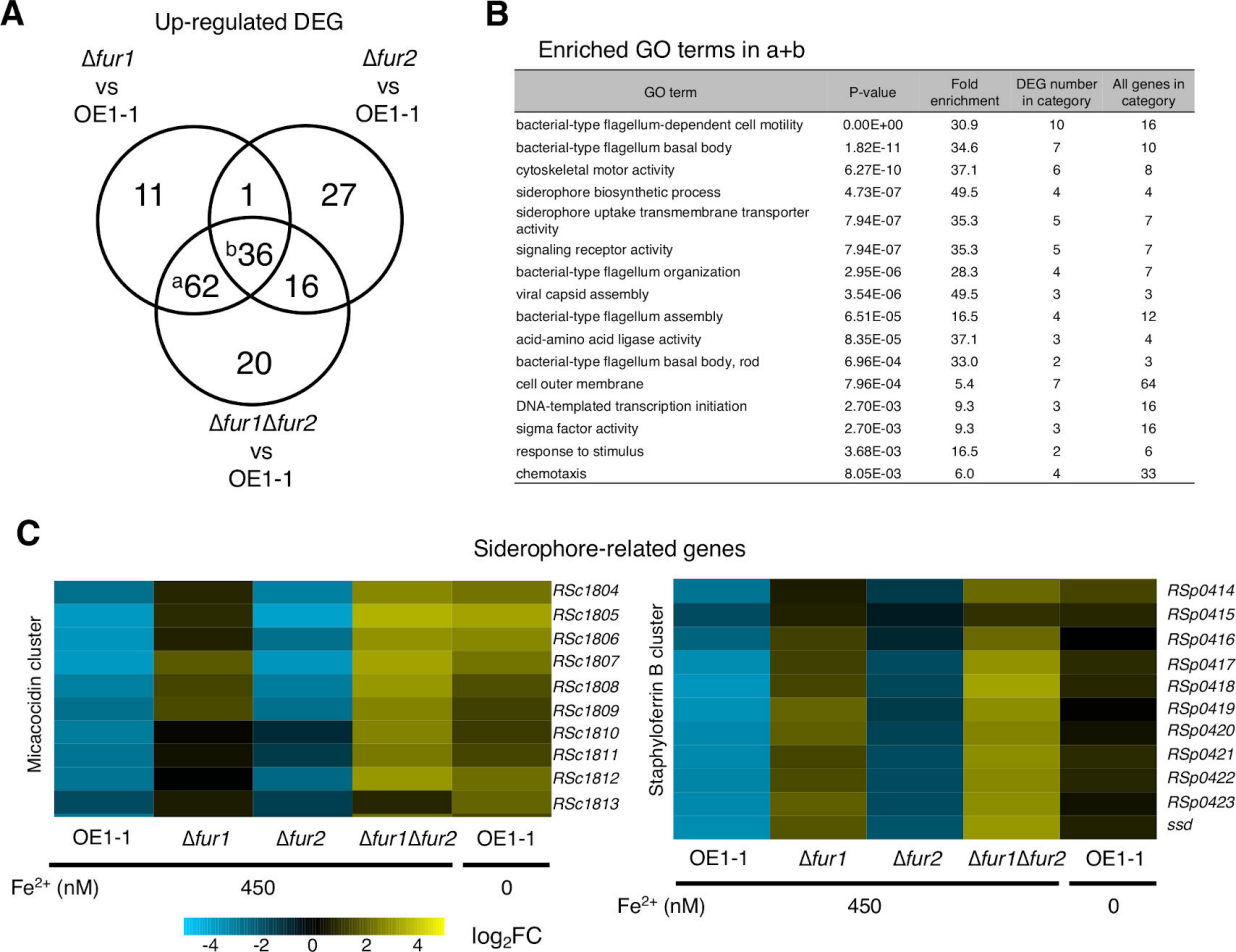
Fur1 and Fur2 cooperatively repress siderophore production-related gene expression in the presence of 450 nM Fe^2+^. (**A**) Venn diagram of the number of differentially expressed genes (DEGs) with a log_2_[∆*fur1*/OE1-1 (450 nM Fe^2+^)] expression level > 2, log_2_[∆*fur2*/OE1-1 (450 nM Fe^2+^)] expression level > 2, and/or log_2_[∆*fur1*Δ*fur2*/OE1-1 (450 nM Fe^2+^)] expression level > 2, with FDR < 0.05. Lowercase letters refer to the gene sets in panel B. (**B**) Enriched Gene Ontology (GO) terms among the upregulated genes indicated by lowercase letters in panel A (*P* < 10^−3^). (**C**) Relative expression levels of genes in two siderophore production-related gene clusters are presented in heatmaps.

Fur proteins have a DNA-binding motif and regulate gene expression in several bacterial species (12). We investigated the potential regulatory effects of Fur1 and Fur2 on gene expression and analyzed the strain OE1-1 transcriptome by completing an RNA sequencing (RNA-seq) analysis. We also generated *fur1* deletion, *fur2* deletion, and *fur1*⁄*fur2* double deletion mutants (∆*fur1*, ∆*fur2*, and ∆*fur1*Δ*fur2*, respectively) in the *R. pseudosolanacearum* strain OE1-1 genetic background. For the RNA-seq analysis, total RNA was extracted from strain OE1-1, ∆*fur1*, ∆*fur2*, and ∆*fur1*Δ*fur2* grown in modified quarter-strength M63 medium supplemented with 450 nM Fe^2+^, as well as from strain OE1-1 grown in medium lacking Fe^2+^ as a control (i.e., Fe^2+^ deficiency). The RNA was extracted from the strains when OD_600_ reached 0.3 during the exponential phase. The Fe^2+^ concentration was selected because quarter-strength M63 medium is generally used as a minimal medium and is likely to supply the sufficient amount of usable iron to *R. pseudosolanacearum* strain OE1-1 (18). Also, it has been reported that *R. solanacearum* can survive in water without iron (19). Read count data for each gene were normalized, and relative expression levels were calculated. Genes with significant differences in expression (|log_2_(fold-change)| > 2, *q*-value < 0.05) relative to the corresponding expression in strain OE1-1 under Fe^2+^-rich conditions (450 nM Fe^2+^) were designated as differentially expressed genes (DEGs). A total of 116, 79, and 138 upregulated DEGs as well as 335, 53, and 398 downregulated DEGs were detected in ∆*fur1*, ∆*fur2*, and ∆*fur1*Δ*fur2*, respectively (Table S1). The distribution of DEGs common to both conditions was analyzed to identify genes controlled by Fur1 and/or Fur2. A Venn diagram analysis showed that many upregulated (98 genes) and downregulated (300 genes) DEGs were shared by ∆*fur1* and ∆*fur1*Δ*fur2*, suggesting that these genes are controlled by Fur1, but are also partially controlled by Fur2 (Fig. 2A).

We performed a Gene Ontology (GO) enrichment analysis to functionally annotate DEGs. The upregulated DEGs shared by ∆*fur1* and ∆*fur1*Δ*fur2* (a and b in Fig. 2A), which are likely controlled by Fur1, were annotated with the GO term “siderophore biosynthetic process” (4/4 genes) (Fig. 2B). The regulatory effects of Fur proteins on siderophore-related genes have been reported for other Gram-negative bacterial species (12), implying that Fur1 is a conserved Fur protein among Gram-negative bacteria. The *R. pseudosolanacearum* genome reportedly includes two gene clusters associated with the production of micacocidin (*RSc1804*–*RSc1813*) and staphyloferrin B (*RSp0414*–*RSp0424*) (13, 14). We analyzed transcriptome data to compare the relative expression levels of these two gene clusters. Under conditions with iron present, the expression levels of both gene clusters increased in ∆*fur1* and ∆*fur1*Δ*fur2*, with greater increases in the double mutant, similar to the corresponding expression in strain OE1-1 under Fe^2+^-deficient conditions. Thus, Fur1 and Fur2 may cooperatively repress the expression of these two siderophore-related gene clusters under Fe^2+^-rich conditions (Fig. 2C). The expression level of some staphyloferrin B cluster genes increased in ∆*fur2,* but that of micacocidin did not, suggesting that Fur2 alone has a repressive effect on staphyloferrin B cluster (Fig. 2C). Under Fe^2+^-rich conditions, Fur1 alone can repress the expression of siderophore-related genes, while Fur2 has repressive effects on these genes in the absence of Fur1 only for micacocidin cluster (Fig. 2B and C). Note that ∆*fur1* and ∆*fur1*Δ*fur2* showed higher expression of staphyloferrin B cluster genes than strain OE1-1 in Fe^2+^ deficiency, suggesting that staphyloferrin B cluster regulation includes both transcription activation and repression: Fur1/2-pathway represses the expression of staphyloferrin B cluster, while other pathway(s) activate it, leading to the balanced expression level (Fig. 2C).

The upregulated DEGs shared by ∆*fur1* and ∆*fur1*Δ*fur2* also included motility-related genes having the GO terms like “bacterial-type flagellum-dependent cell motility,” “bacterial-type flagellum basal body,” “bacterial-type flagellum organization,” “bacterial-type flagellum assembly,” “bacterial-type flagellum basal body, rod,” and “chemotaxis” (Fig. 2B). The terms related to flagella are also enriched in the DEGs only in ∆*fur2* (b in Fig. S3A and C), ∆*fur2* and ∆*fur1*Δ*fur2* (d), and ∆*fur1*, ∆*fur2*, and ∆*fur1*Δ*fur2* (f), suggesting that Fur1 and Fur2 contribute to the regulation of flagellum-related genes in cooperative manners (Fig. S3A and C). It was also found that virulence-related genes, such as *eps* operon and endo-1,4-β-glucanase-encoding *egl*, were downregulated significantly in ∆*fur1* and ∆*fur1*Δ*fur2* and moderately in strain OE1-1 under iron-deficient conditions (Table S1). This result indicates that the expression of virulence-related genes is induced by Fur1 under the presence of Fe^2+^. Overall, Fur1 and Fur2 also have pleiotropic regulatory effects on various genes other than the siderophore-related genes in strain OE1-1.

### Fur1 is the main regulator of extracellular Fe^3+^-chelating activity under Fe^2+^-rich conditions, but Fur2 is also involved

**Fig 3:**
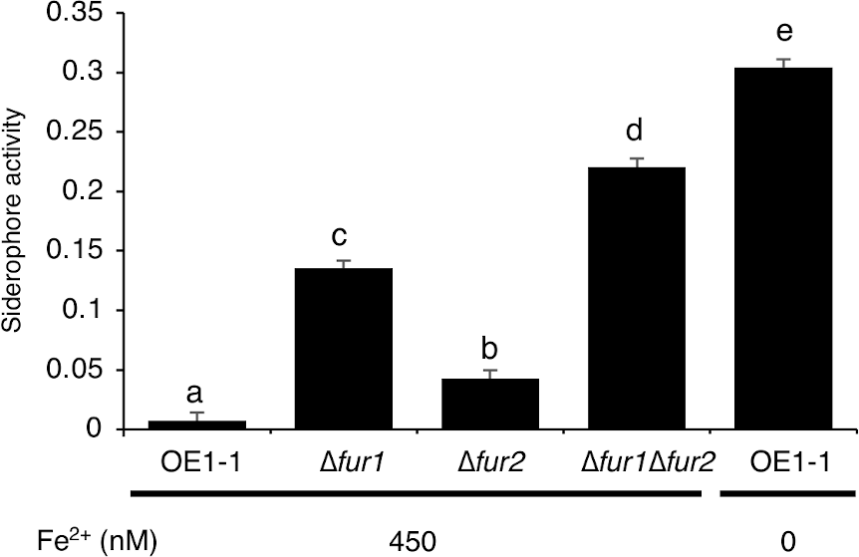
Low extracellular Fe^3+^-chelating activity in the presence of 450 nM Fe^2+^ is mainly maintained by Fur1, with assistance from Fur2. Mean Fe^3+^-chelating activity levels [(A_630 Control_ – A_630 Sample_)/A_630 Control_] of different samples are presented. Fe^2+^ concentrations in the growth medium are indicated at the bottom. Strain OE1-1 grown under 0 nM Fe^2+^ conditions was used as a positive control. Eight biological replicates were used (standard deviations are presented as error bars). Mean extracellular Fe^3+^-chelating activity levels were analyzed for significant differences between *R. pseudosolanacearum* strains via analysis of variance followed by Tukey–Kramer’s honestly significant difference test. Significant differences are indicated by different lowercase letters (*P* < 0.01).

In certain bacterial species, Fur proteins control extracellular Fe^3+^-chelating activity, which is repressed in the presence of sufficient iron and de-repressed in response to iron deficiency. To determine whether Fur1 and Fur2 control extracellular Fe^3+^-chelating activity, an *in vitro* extracellular Fe^3+^-chelating activity assay (18, 20) was conducted using ∆*fur1*, ∆*fur2*, and ∆*fur1*Δ*fur2* as well as strain OE1-1. The extracellular Fe^3+^-chelating activity of strain OE1-1 under Fe^2+^-rich conditions was lower than that in the absence of Fe^2+^ (Fig. 3). The extracellular Fe^3+^-chelating activity was higher for ∆*fur1* and ∆*fur2* than for strain OE1-1 under Fe^2+^-rich conditions, with higher activity detected for ∆*fur1* than for ∆*fur2* (Fig. 3). Notably, the extracellular Fe^3+^-chelating activity was higher for ∆*fur1*Δ*fur2* than for ∆*fur1* and ∆*fur2* under Fe^2+^-rich conditions, suggesting that Fur1 and Fur2 redundantly repress the extracellular Fe^3+^-chelating activity under Fe^2+^-rich conditions (Fig. 3). However, Fur1 seems to have a stronger repressive effect than Fur2.

### Fur1 and Fur2 redundantly affect the expression of various metabolism-related genes

Our transcriptome analysis also revealed the redundant regulatory effects of Fur1 and Fur2 on gene expression. A total of 20 and 102 genes were identified as upregulated and downregulated DEGs, respectively, only in ∆*fur1*Δ*fur2* under Fe^2+^-rich conditions (Fig. 2A). Accordingly, Fur1 and Fur2 may redundantly regulate the expression of these genes under Fe^2+^-rich conditions (Fig. 2A; Fig. S3A and B). Enriched GO terms among DEGs specific to ∆*fur1*Δ*fur2* were related to nitrate metabolism. More specifically, “nitrate reductase activity” (4/5 genes) and “nitrate assimilation” (4/8 genes) were GO terms assigned to the upregulated DEGs exclusive to ∆*fur1*Δ*fur2*. Hence, Fur1 and Fur2 redundantly repress the expression of these nitrate metabolism-related genes (Fig. S3C). The GO terms “integral component of membrane” (42/1,164 genes) and “oxidoreductase activity” (7/112 genes) were enriched among the downregulated DEGs; however, the specific functions of these genes in terms of bacterial metabolism were not obvious because the enrichment was not strong (Fig. S3D).

### Deleting *fur2* decreased growth, but deleting both *fur1* and *fur2* resulted in a much greater decrease

**Fig 4:**
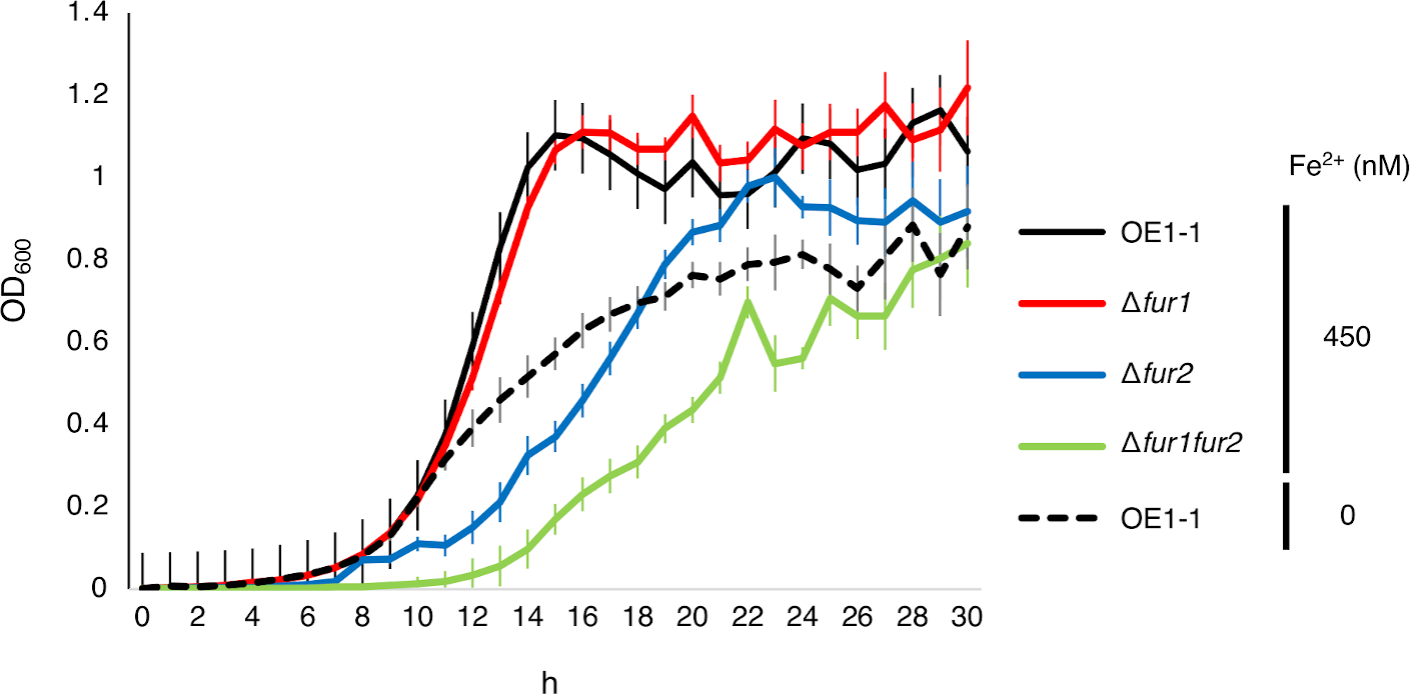
Deleting *fur2* alone and deleting both *fur1* and *fur2* decreased the *in vitro* growth level. Strain OE1-1, ∆*fur1*, and ∆*fur2* growth levels at 30°C under aerobic conditions were monitored hourly. Seven biological replicates per strain were grown under the indicated Fe^2+^ conditions. Averages and standard error bars are presented.

According to the transcriptome analysis, Fur1 and Fur2 affect the expression of various metabolism-related genes and siderophore production-related genes. We also analyzed the effects of Fur1 and Fur2 on the growth levels in response to different extracellular Fe^2+^ levels. Bacterial growth (per hour) was measured based on the optical density at 600 nm (OD_600_) values of cultures of strain OE1-1, ∆*fur1*, ∆*fur2*, and ∆*fur1*Δ*fur2*. The bacterial growth of ∆*fur2* was lower than that of strain OE1-1, whereas ∆*fur1* had a similar growth level to that of strain OE1-1 (Fig. 4). Notably, bacterial growth was much lower for ∆*fur1*Δ*fur2* than for ∆*fur2*, suggesting that Fur1 and Fur2 redundantly affect the growth of strain OE1-1 (Fig. 4). The early growth level of strain OE1-1 in medium without Fe^2+^ was the same as that of strain OE1-1 in medium with 450 nM Fe^2+^, but after that, the growth slowed down as a result of iron deficiency (Fig. 4). The observed bacterial growth indicated that Fur2 may be needed to maintain an appropriate strain OE1-1 growth level, but Fur1 has redundant effects.

### Fur1 and Fur2 contribute to the full virulence of OE1-1 in tomato plants

**Fig 5:**
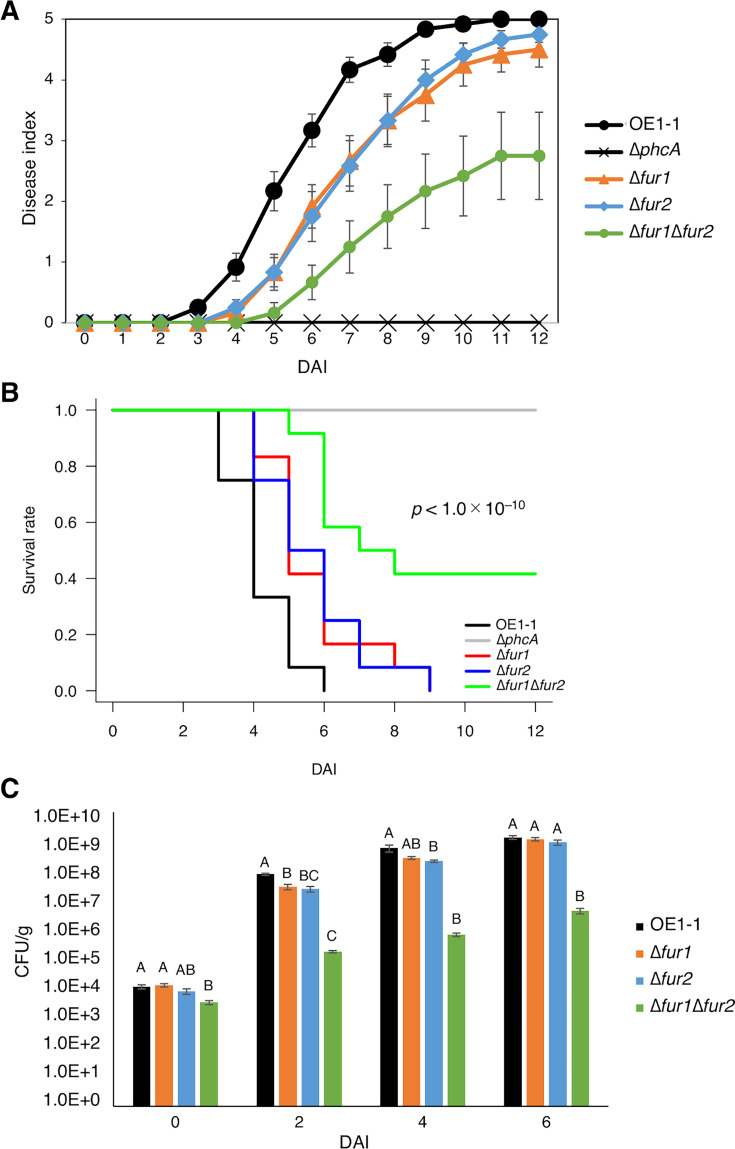
Fur1 and Fur2 cooperatively maintain the full virulence of strains in tomato plants. (**A**) Virulence of *R. pseudosolanacearum* strains. Inoculated plants were examined using the following disease index scale: 0, no wilting; 1, 1%–25% wilting; 2, 26%–50% wilting; 3, 51%–75% wilting; 4, 76%–99% wilting; and 5, dead. For each bacterial strain, six replicate plants were analyzed twice. Data were combined and presented as the mean ± SE of 12 replicates. (**B**) Survival rates of plants inoculated with *R. pseudosolanacearum* strains, with the Chi-squared test conducted to determine *P*-values. (**C**) *R. solanacearum* strain populations in the roots of tomato plants inoculated using a root-dipping method were analyzed using Hara–Ono medium. CFU, colony-forming unit. SE is indicated by an error bar. Mean abundances were analyzed for significant differences between *R. pseudosolanacearum* strains via an analysis of variance followed by Tukey–Kramer’s honestly significant difference test. Significant differences are indicated by different lowercase letters (*P* < 0.05).

Fur family members are important for the virulence of many bacterial pathogens (12). To clarify the effects of Fur1 and Fur2 on virulence, we conducted virulence assays involving tomato plants, with a previously reported non-pathogenic strain (∆*phcA*) serving as a negative control (20–22). The disease index was markedly lower for plants inoculated with either ∆*fur1* or ∆*fur2* than for plants inoculated with strain OE1-1 at 4–7 days after inoculation (DAI), reflecting the effects of Fur1 and Fur2 on the virulence of OE1-1 (Fig. 5A). Notably, the disease index was lower for plants inoculated with ∆*fur1*Δ*fur2* than for plants inoculated with ∆*fur1* and/or ∆*fur2* at 6 DAI (Fig. 5A). Furthermore, the survival rate of plants inoculated with ∆*fur1* and ∆*fur2* decreased more slowly than that of plants inoculated with strain OE1-1, while that of plants inoculated with ∆*fur1*Δ*fur2* decreased much more slowly, with five of 12 ∆*fur1*Δ*fur2*-inoculated plants surviving until 12 DAI (Fig. 5B).

We also analyzed *R. pseudosolanacearum* strain populations in tomato plant roots. At 2 DAI, ∆*fur1* and ∆*fur2* were significantly less abundant than strain OE1-1 in tomato roots (*P* < 0.05) (Fig. 5C). Additionally, ∆*fur1*Δ*fur2* was considerably less abundant than ∆*fur1* and ∆*fur2* at 6 DAI (*P* < 0.05). Note that ∆*fur1*Δ*fur2* showed a significant decrease in the population even at 0 DAI, which is possibly because of the lower attachment rate in tomato plant roots. Accordingly, Fur1 and Fur2 cooperatively maintain the bacterial population (Fig. 5C). The observed decrease in the disease index and the slow decrease in the survival rate of plants inoculated with ∆*fur1* and ∆*fur2* imply that both Fur1 and Fur2 contribute to the virulence of strain OE1-1. Furthermore, based on the results for ∆*fur1*Δ*fur2*, Fur1 and Fur2 likely cooperatively influence the virulence of strain OE1-1.

## DISCUSSION

Considering that RSSC exists under both iron-limited conditions in rhizosphere soil and iron-rich conditions within host plants, the mechanism through which bacterial cells perceive and respond to extracellular iron conditions must be thoroughly characterized (2, 3, 23). In the current study, we revealed that RSSC has two Fur proteins (Fur1 and Fur2). Furthermore, a transcriptome analysis indicated that Fur1 and Fur2 cooperatively control siderophore-related gene expression (Fig. 2). Deleting *fur1* alone leads to increased siderophore-related gene expression, which is repressed under Fe^2+^-rich conditions, but deleting both *fur1* and *fur2* results in even higher siderophore-related gene expression (Fig. 2C). Thus, Fur1 may be a major repressor of siderophore-related gene expression, with Fur2 providing secondary repressive effects. The rank order of extracellular Fe^3+^-chelating activity among *R. pseudosolanacearum* strains under Fe^2+^-rich conditions was as follows: OE1-1 < ∆*fur2* < ∆*fur1* < ∆*fur1*Δ*fur2* (Fig. 3). Therefore, Fur1 and Fur2 may cooperatively maintain low extracellular Fe^3+^-chelating activities under Fe^2+^-rich conditions by repressing siderophore-related gene expression in RSSC. In addition, deleting both *fur1* and *fur2* decreased the expression of nitrate metabolism-related genes, which was in contrast to the lack of significant changes following the deletion of *fur1 *or *fur2* (i.e., single deletions). These results suggest that Fur1 and Fur2 redundantly control the expression of nitrate metabolism-related genes (Fig. S3A and C). *R. solanacearum* exploits nitrogen metabolism for respiration in addition to oxygen metabolism under the low-oxygen conditions typically found in xylem vessels (24). The deletion of denitrification-related genes alters iron-related gene expression, indicating that denitrification influences iron metabolism (25). Thus, nitrogen metabolism and iron-dependent gene regulation are tightly linked.

The transcriptome analysis in this study also revealed that Fur1 and Fur2 have pleiotropic regulatory effects on various genes. It was revealed that Fur1 and Fur2 cooperatively contribute to the repression of motility-related genes involved in flagellum assembly or chemotaxis (Fig. 2B; Fig. S3A and C). The transcriptome data showed that strain OE1-1 under the iron-deficient condition did not show the increased expression of flagellum components such as *flg* genes, suggesting that there are also induction pathways dependent on the presence of Fe^2+^, in addition to repression pathways by Fur1 and Fur2 (Table S1). The transcriptome analysis also revealed that some virulence genes, including *eps* operon and *egl*, are upregulated by Fur1 (Table S1). It has been reported using *Escherichia coli* that Fur also has direct inducive effects on some genes, as well as repressive effects (26). Although it is unclear whether the virulence genes such as *eps* operon and *egl* are directly regulated by Fur1, it is possible that Fur1 and Fur2 have inducive effects on target gene expression in strain OE1-1.

An analysis of virulence in tomato plants showed that deleting *fur1 *or *fur2* decreased virulence, but deleting both *fur1* and *fur2* resulted in an even greater decrease (Fig. 5A and B). Furthermore, we could observe the decrease of bacterial population and virulence on tomato plants in ∆*fur1*Δ*fur2* compared to ∆*fur1* and ∆*fur2* (Fig. 5C). In the case of ∆*fur1* and ∆*fur2*, the bacterial population decreased at 2 DAI but recovered to the same level as that in strain OE1-1 by 6 DAI, while the disease index also became close to that in strain OE1-1 by 12 DAI, suggesting that the single deletion caused the delay in both population increase and disease progression (Fig. 5). On the other hand, the bacterial population increase and the disease progression of ∆*fur1*Δ*fur2* did not reach the same level of those in strain OE1-1, suggesting that Fur1 and Fur2 have cooperative effects on the maintenance of both bacterial population and virulence during infection (Fig. 5). However, it is still unclear how the virulence of ∆*fur1*Δ*fur2* decreased in tomato plants because the virulence of *R. pseudosonalacearum* is likely determined as a result of interaction with host plants. It is possible that ∆*fur1*Δ*fur2* did not reach the threshold of population to accomplish the full virulence, or that ∆*fur1*Δ*fur2*’s phenotypic changes reduced its virulence. In addition, an increase in extracellular Fe^3+^-chelating activity possibly explains the decreased virulence of ∆*fur1*, ∆*fur2*, and ∆*fur1*Δ*fur2* (Fig. 5A and B). Earlier research demonstrated that host plants and microbes compete for available iron (27, 28). A previous *in planta* transcriptome analysis of *Pseudomonas syringae* in *Arabidopsis thaliana* leaves indicated that an iron acquisition pathway is a major target of plant immunity-related mechanisms (29). It is possible that the increased Fe^3+^-chelating activities of ∆*fur1* and ∆*fur1*Δ*fur2* lead to induced plant immunity. Additionally, excessive amounts of Fe^2+^ may be harmful to bacteria because the Fenton reaction produces hydroxyl radicals in the presence of free Fe^2+^ (8). Excessive intracellular Fe^2+^ levels in ∆*fur1* and ∆*fur1*Δ*fur2* may lead to decreased virulence. However, further studies are needed to address the effect of inner host plant environment on the bacteria and the bacterial behaviors in response to it. These studies will also address the mechanisms by which the virulence of *fur1*- and/or *fur2*-deletion strains resulted in the delay and/or decrease of virulence.

A simple working model for Fur proteins has been developed. Specifically, homodimeric Fe^2+^-binding Fur proteins bind to a cis-element in the target promoter to prevent the binding of RNA polymerase (12). On the basis of the transcriptome data in the current study, both Fur1 and Fur2 may function as repressors (Fig. 2C). In the case of single deletion, the effect of Fur1 was stronger than that of Fur2; however, considering the case of double deletion, Fur2 was also important for siderophore regulation, and there seems to be mechanisms controlling siderophore production through the cooperation of Fur1 and Fur2. The mechanism is unknown, and further biochemical analyses are needed (Fig. 2C). According to our phylogenetic analysis, Fur1 and Fur2 belong to different groups (Fig. 1; Fig. S2). Fur1 has a motif structure that is conserved among Fur proteins in closely related non-RSSC *Burkholderia* species, whereas Fur2 appears to be a different clade protein with a different C-terminal motif sequence and the predicted structure, suggesting that Fur1 and Fur2 are possibly paralogs with different functions (Fig. 1). The presence of two Fur proteins is a characteristic feature of RSSC strains and possibly mediates the virulence of RSSC. However, differences in the mechanisms underlying Fur1 and Fur2 functions will need to be determined in future studies.

## MATERIALS AND METHODS

### Bacterial strains, plasmids, and growth conditions

Bacterial strains used in this study are listed in Table S2. All *R. pseudosolanacearum* strains were routinely grown at 30°C in a modified quarter-strength M63 medium [3.75 mM (NH_4_)_2_SO_4_, 667 µM MgSO_4_, 25 mM KH_2_PO_4_, and 450 nM FeSO_4_] with 20 mM sodium L-glutamate as the carbon source. For control growth conditions (i.e., without Fe^2+^), quarter-strength M63 medium was prepared without 450 nM FeSO_4_. For the siderophore activity assay and growth analysis, 450 nM FeSO_4_ was replaced by 450 nM FeCl_2_. *E. coli* strains were grown at 37°C in lysogeny broth medium (30). To determine the growth level of *R. pseudosolanacearum* strains, the OD_600_ value was recorded hourly using a TVS062CA Biophotorecorder (Advantec, Tokyo, Japan).

### Phylogenetic analysis

Putative Fur amino acid sequences were obtained from the NCBI database following a BLASTP search using *R. pseudosolanacearum* strain OE1-1 Fur1 and Fur2 protein sequences as queries. Protein sequences in RSSC strains for all four phylotypes and closely related species (31) were used as references. Finally, we obtained eight sequences from RSSC strains and 14 sequences from closely related species. Additionally, the Fur protein in *E. coli* strain K-12 served as an outgroup for the phylogenetic analysis, which was performed using MEGA11 (32). The MUSCLE program was used for aligning multiple sequences. A phylogenetic tree was constructed according to the maximum likelihood method, with 1,000 bootstrap replicates and the following options: Test of Phylogeny: None; Model/Method: WAG model; Rates Among Sites: Gamma distributed with invariant sites (G+I); Number of Discrete Gamma Categories: 5; Gaps/Missing Data Treatment: Complete deletion; ML Heuristic Method: Nearest-neighbor interchange; Initial Tree for ML: Make initial tree automatically; Branch Swap Filter: Very strong. To compare domain structures, the Clustal Omega web server of EMBL was used to align multiple sequences (https://www.ebi.ac.uk/Tools/msa/clustalo/). Previously determined domain structures (16) were used for the analysis. To obtain the identity scores of full-length amino acid sequences, a FASTA file was first generated using the Clustal Omega web server, with Fur1 and Fur2 amino acid sequences from all four RSSC phylotypes as inputs. The FASTA file was analyzed using the Ident and Sim function of the Sequence Manipulation Suite (https://www.bioinformatics.org/sms2/ident_sim.html) to calculate the amino acid sequence identity (33).

### Conserved motif analysis

Fur protein amino acid sequences used for the phylogenetic analysis were screened for conserved motifs using MEME (version 5.5.7; https://meme-suite.org/meme/tools/meme), with the number of motifs set to 10 (34).

### Prediction of protein structures by AlphaFold 3

Structures of Fur1 and Fur2 were predicted using the online AlphaFold server with AlphaFold 3 (https://alphafoldserver.com/; [35]) by inputting each amino acid sequence of Fur1 and Fur2 with default parameters. The predicted models were colored with the same colors of the MEME analysis outputs and represented by ChimeraX (36).

### General DNA manipulations

Genomic DNA was isolated, plasmid DNA was manipulated, and PCR amplifications were completed using standard techniques (37). *R. pseudosolanacearum* strain OE1-1 was transformed via electroporation (38). Double-stranded DNA sequencing templates were prepared using the GenElute Plasmid Miniprep Kit (Sigma Chemical Co., St. Louis, MO, USA). Sequences were determined using the ABI Prism 3100-Avant Genetic Analyzer (Applied Biosystems, Tokyo, Japan). DNA sequencing data were analyzed using DNASIS-Mac software (Hitachi Software Engineering, Yokohama, Japan).

### Generation of *fur1* and *fur2* deletion mutants

Plasmids used in this study are listed in Table S2. A 697 bp DNA fragment (delta-fur1-1) was amplified by PCR using strain OE1-1 genomic DNA (NCBI Reference Sequence: NZ_CP009764.1) as the template and primers delta-fur1-1-FW (5′-CGgga tccAC GGAAT CGACG GAGCG C-3′), which includes a BamHI site (lowercase letters), and delta-fur1-1-RV (5′-CTGTT GCGTC ACATG CGCCT GGCTC CCTA-3′). A 706 bp DNA fragment (delta-fur1-2) was amplified by PCR using strain OE1-1 genomic DNA and primers delta-fur1-2-FW (5′-AGGCG CATGT GACGC AACAG CCGCC TCAT-3′) and delta-fur1-2-RV (5′-CCCaa gcttA GCTGG ACCTG GCGTT CTC-3′) with a HindIII site (lowercase letters). Amplified delta-fur1-1 and delta-fur1-2 sequences, along with primers delta-fur1-1-FW and delta-fur1-2-RV, were used for a PCR amplification of a 1,384 bp DNA fragment, which was then digested with BamHI (Takara Bio, Ohtsu, Japan) and HindIII (Takara Bio). The resulting 1.4 kb fragment was inserted into the pK18mobsacB vector digested with BamHI and HindIII (39) to produce the pdelta-*fur1* recombinant plasmid. A 769 bp DNA fragment (delta-fur2-1) was amplified by PCR using strain OE1-1 genomic DNA and primers delta-fur2-1-FW (5′-CGgga tccAA AAGGT CAACG GCCTG CGC-3′), which includes a BamHI site (lowercase letters), and delta-fur2-1-RV (5′-TGCAA CGCCT ACATG GTGTT GGGGC GAGAA G-3′). A 706 bp DNA fragment (delta-fur2-2) was amplified by PCR using strain OE1-1 genomic DNA and primers delta-fur2-2-FW (5′-CCAAC ACCAT GTAGG CGTTG CACGC GCTTC A-3′) and delta-fur2-2-RV (5′-CCCaa gcttG AGAAG GAAGA CCCGG TGG-3′) with a HindIII site (lowercase letters). Amplified delta-fur2-1 and delta-fur2-2 sequences, along with primers delta-fur2-1-FW and delta-fur2-2-RV, were used for PCR amplification of a 1,444 bp DNA fragment, which was then digested with BamHI and HindIII. The obtained 1.4 kb fragment was inserted into the pK18mobsacB vector, digested with BamHI and HindIII, to produce the pdelta-*fur2* recombinant plasmid. An electroporation-based method was used to insert recombinant plasmids into strain OE1-1 competent cells, which were prepared as previously described (40). Kanamycin-sensitive, sucrose-resistant recombinants, Δ*fur1* or ∆*fur2* (Table S2), were selected. To verify the deletion of *fur1 *(*RSc2747*), a 1,636 bp DNA fragment (WT: 2,061 bp) was amplified by PCR using primers delta-fur1-SQ-FW (5′-CTCGC TGGTG GTCAT GCTG-3′) and delta-fur1-SQ-RV (5′-GCTTC CTGCT GCGCG TC-3′). The fragment sequence was analyzed using primers delta-fur1-SQ-FW and delta-fur1-SQ-RV. To confirm *fur2 *(*RSp0247*) was deleted, a 1,826 bp DNA fragment (WT: 2,276 bp) was amplified by PCR using primers delta-fur2-SQ-FW (5′-ATGGT CCGCT GTTCA AGGGC-3′) and delta-fur2-SQ-RV (5′-TGGCC GAGCA GCCGA TC-3′). The fragment sequence was analyzed using primers delta-fur2-SQ-FW and delta-fur2-SQ-RV. To delete both *fur1* and *fur2*, *fur2* was deleted from ∆*fur1*.

### RNA-seq

*R. pseudosolanacearum* strains were grown in quarter-strength M63 medium or modified quarter-strength M63 medium without Fe^2+^ until the OD_600_ value reached 0.3. The growth level was checked in a timely manner by recording OD_600_ hourly using a TVS062CA Biophotorecorder (Advantec, Tokyo, Japan) in the same way as bacterial growth levels were analyzed. Total RNA was extracted using a High Pure RNA Isolation kit (Roche Diagnostics, Mannheim, Germany). Ribosomal RNA was eliminated from the extracted RNA using a Ribo-Zero rRNA Removal kit (Gram-negative bacteria) (Illumina, Madison, WI, USA), as previously described (41). An oriented, paired-end RNA-seq analysis (2 × 100 bp) was performed using an Illumina HiSeq 2500 system or a DNBSEQ-G400 system. Generated reads were trimmed using Cutadapt (version 1.1; http://code.google.com/p/cutadapt/ [42]) and then mapped to the GMI1000 strain genome using Bowtie2 (version 2.4.2) (43). Read count data were obtained using featureCounts (version 2.0.0) (44). Three independent biological replicates were analyzed per strain.

### Data analysis

RNA-seq data were analyzed using R cran (45). Genes with no RNA-seq reads in at least one sample in the raw count data set were excluded. RNA-seq read counts of the remaining genes were normalized using calcNormFactors (trimmed mean of M-values normalization) in edgeR (46). To extract genes with significant changes in transcript levels, the following thresholds were applied: *q*-value < 0.05 and |log_2_(fold-change)| ≥ 2. FDR (*q*-value) was calculated using *P*-values estimated by edgeR according to the Benjamini–Hochberg method (47). Heatmaps were created using the R package pheatmap.

### Gene ontology enrichment analysis

A GO enrichment analysis was performed using the R package GoSeq (48), with GO terms obtained from QuickGO (https://www. ebi.ac.uk/Quick GO/). Fold enrichment was calculated as follows: (number of DEGs annotated with the term/number of all genes annotated with the term)/(number of DEGs/number of all genes).

### Extracellular Fe^3+^-chelating activity analysis

The extracellular Fe^3+^-chelating activity of *R. pseudosolanacearum* strains was analyzed as previously described (18, 20). Briefly, *R. pseudosolanacearum* strains were grown at 30°C in modified quarter-strength M63 medium containing 450 nM FeCl_2_ or 0 nM FeCl_2_ (control) for 18 h, and then the culture concentration was adjusted to 2.0 × 10^9^ CFU/mL using 0.1 M PIPES buffer (pH 6.5). After a 6-hour incubation, each culture was passed through a 0.2 μm pore filter. Next, a 100 µL aliquot of each culture or PIPES buffer (i.e., reference) was added to 100 μL chromazurol S (CAS) solution (2.4 mM hexadecyl-trimethyl ammonium bromide, 0.06 mM FeCl_3_, 0.6 mM HCl, and 0.6 mM CAS in PIPES buffer). After a 30-minute incubation at 30°C, the absorbance at 630 nm was measured. To calculate the Fe^3+^-chelating activity, the absorbance of the reference was subtracted from the total absorbance. Seven or eight biological replicates were used per assay. Mean values were analyzed by analysis of variance with Tukey–Kramer’s honestly significant difference test to assess the significance of any differences among *R. pseudosolanacearum* strains (strain OE1-1 as the reference).

### Virulence assay

Three-week-old tomato plants (*Solanum lycopersicum* ‘Ohgata-Fukuju’) were inoculated with *R. pseudosolanacearum* strains (1.0 × 10^8^ CFU/mL) using a published root-dip method (49). Plants were monitored daily for wilting symptoms, which were rated according to the following disease index scale: 0, no wilting; 1, 1%–25% wilting; 2, 26%–50% wilting; 3, 51%–75% wilting; 4, 76%–99% wilting; and 5, dead. For each bacterial strain, two independent groups were tested, with six biological replicates per group.

## Data Availability

Data supporting the study findings are available from the corresponding author upon reasonable request. The raw read data used for the transcriptome analysis are available in the Short Read Archive of the GEO database under the following accession numbers: PRJDB15155 (DRR438005, DRR43806, and DRR438007), PRJDB16234 (DRR493625 and DRR493446), and PRJDB17467.

## SUPPLEMENTAL MATERIAL

Supplemental figures aem.02276-25-s0001.pdf Figures S1 to S3.

Table S1 aem.02276-25-s0002.xlsx RNA sequencing data.

Table S2 aem.02276-25-s0003.xlsx Bacterial strains, plasmids, and primers used in this study.

ASM does not own the copyrights to Supplemental Material that may be linked to, or accessed through, an article. The authors have granted ASM a non-exclusive, world-wide license to publish the Supplemental Material files. Please contact the corresponding author directly for reuse.
