# Supplementary material for: Two ferric uptake regulator proteins in Ralstonia pseudosolanacearum strain OE1-1 function cooperatively in response to the extracellular iron level under ferrous iron-rich conditions
Source: Appl Environ Microbiol. 2026 Mar 2;92(3):e02276-25. doi: 10.1128/aem.02276-25 (PMC12997786; doi:10.1128/aem.02276-25)
Supplement: Supplemental figures — Figures S1 to S3. [file aem.02276-25-s0001.pdf]

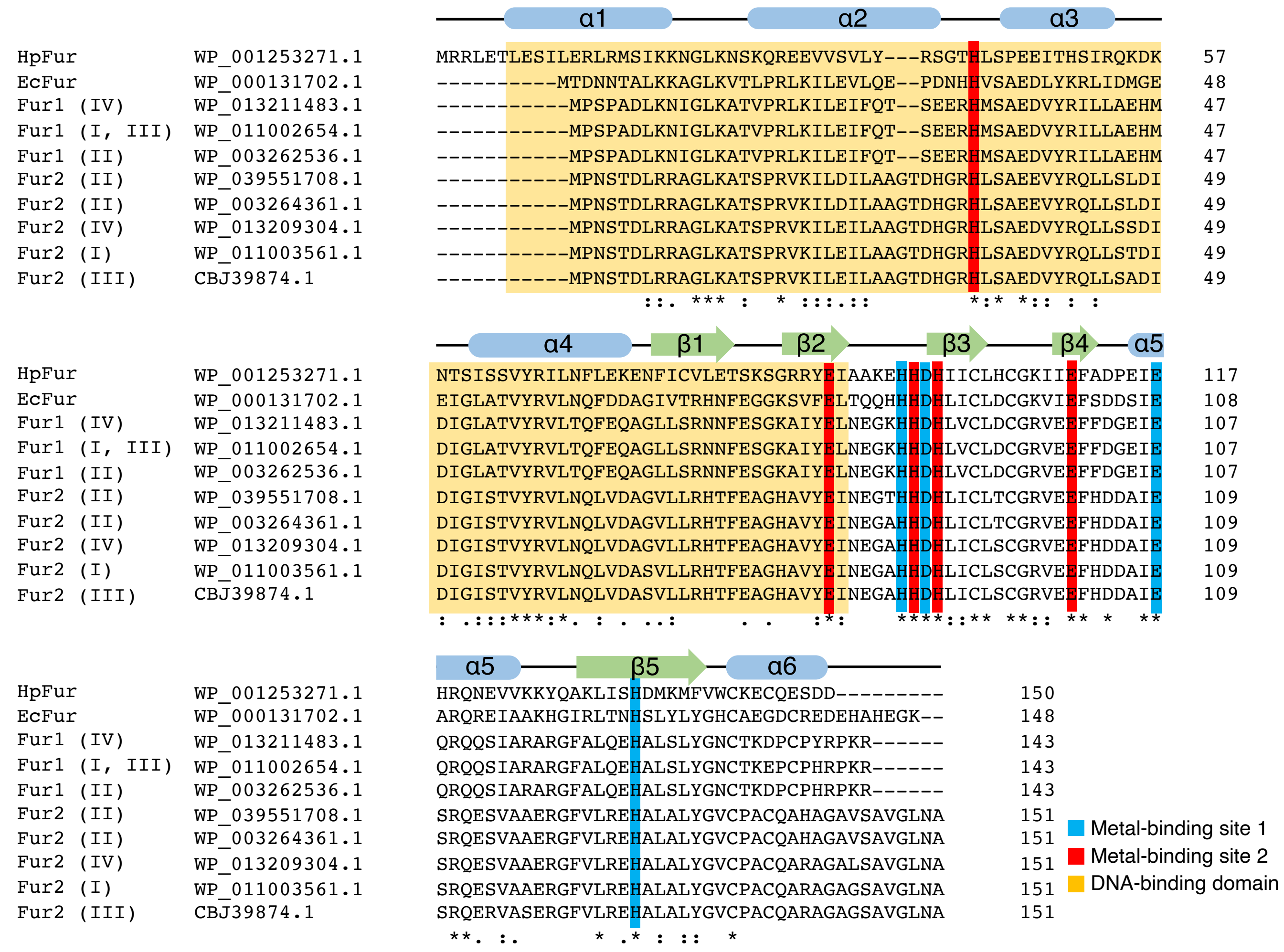

Fig. S1 Multiple alignment of two Ferric uptake regulator paralogs in RSSC.

The alignment of amino acid sequences of multiple Fur proteins with their IDs. Fur in *Escherichia coli* (EcFur) and in *Helicobacter pylori* (HpFur) were included as references. For the sequences in RSSC species, the phylotype number is described. Fur1 and Fur2 in the OE1-1 strain. Domain structures determined in Dian et al (2011) are shown above the alignment. Corresponding amino acids for putative two metal-binding sites 1 (blue shaded), 2 (red shaded) and DNA-binding domain (yellow shaded) are shown on the alignment.

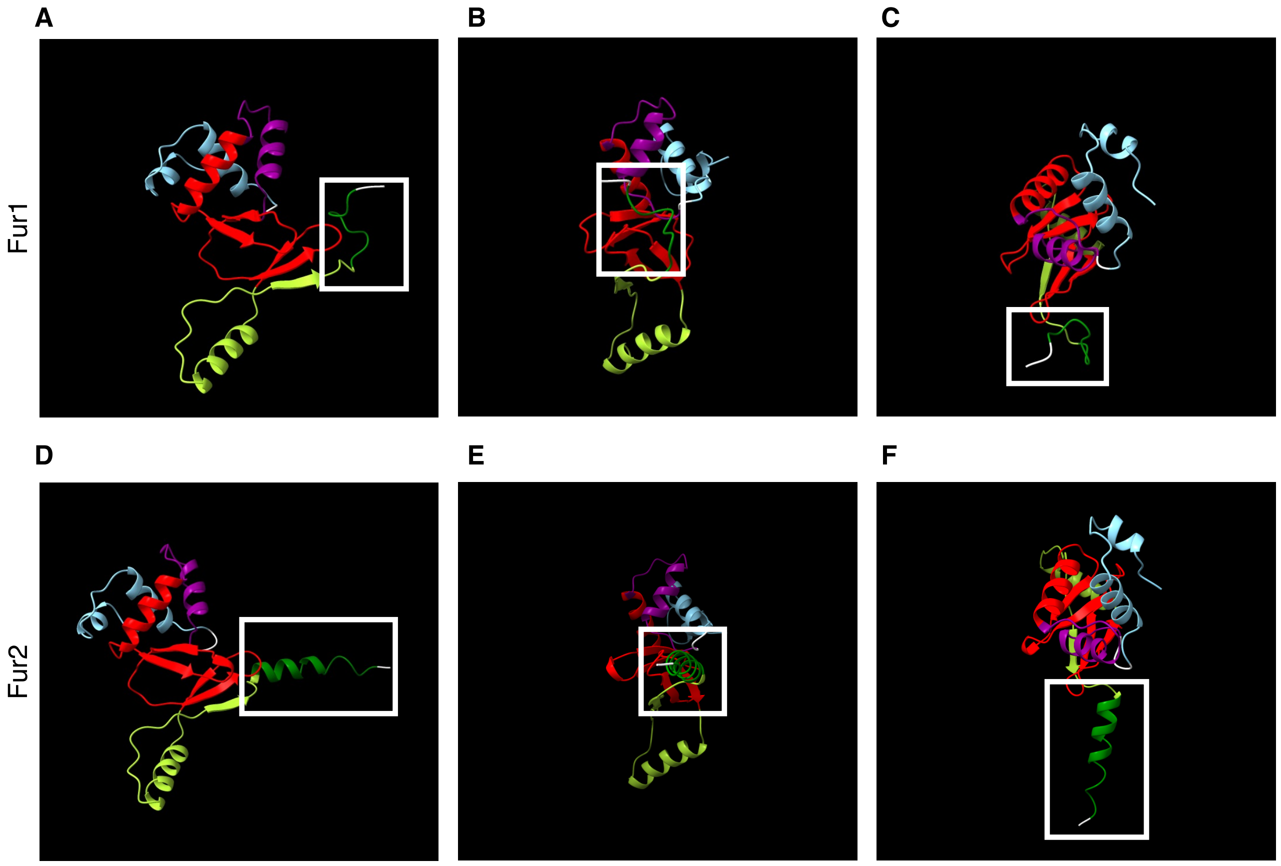

Fig. S2 AlphaFold 3 models of Fur1 and Fur2

(A–F) Ribbon models of Fur1 (A–C) and Fur2 (D–F) predicted by AlphaFold 3 are represented. Ribbon colors were same as those predicted as conserved motif sequences by MEME analysis shown in Fig. 1A and B. White rectangles indicate the C-terminal structures of Fur1 or Fur2.

A

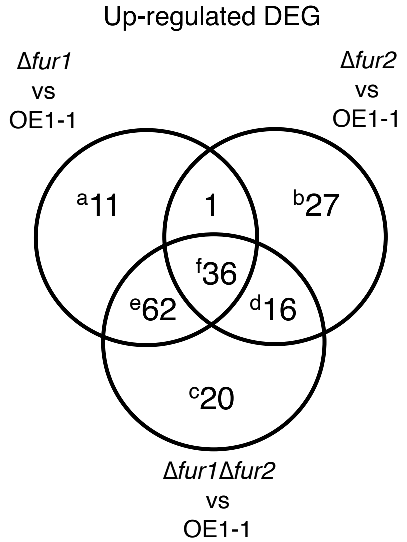

B

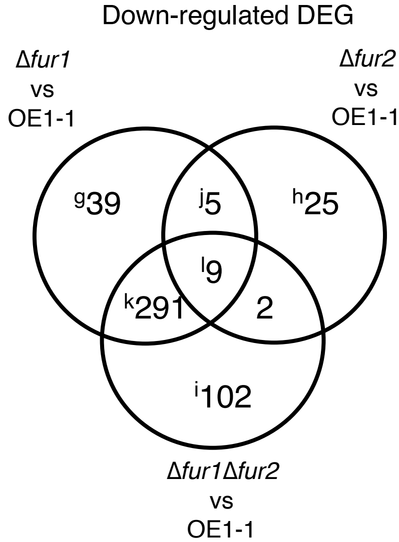

C

#	GO term	P value	Fold enrichment	DEG number in category	All genes in category
	cytochrome-c oxidase activity	1.2E-05	73.44	3	18
	oxidoreductase activity, acting on diphenols and related substances as donors, oxygen as acceptor	4.1E-05	220.32	2	4
	cytochrome bo3 ubiquinol oxidase activity	9.6E-05	146.88	2	6
a	respirasome	1.4E-04	125.90	2	7
	integral component of membrane	2.3E-04	3.41	9	1164
	tRNA 5-leader removal	2.6E-03	440.64	1	1
	ribonuclease P activity	2.6E-03	440.64	1	1
	chemotaxis	0.0E+00	59.84	11	33
	signal transduction	7.8E-10	48.33	7	26
	transmembrane signaling receptor activity	4.3E-07	47.24	5	19
	protein-glutamine glutaminase activity	2.6E-05	179.52	2	2
	archaeal or bacterial-type flagellum-dependent cell motility	6.1E-05	119.68	2	3
	bacterial-type flagellum	3.0E-04	59.84	2	6
	structural molecule activity	5.8E-04	59.84	2	6
b	phosphorelay signal transduction system	2.5E-03	9.29	3	58
	protein-glutamate O-methyltransferase activity	4.9E-03	179.52	1	1
	extracellular region	5.6E-03	22.44	2	16
	protein demethylation	5.9E-03	179.52	1	1
	protein-glutamate methylesterase activity	5.9E-03	179.52	1	1
	protein deamination	5.9E-03	179.52	1	1
	regulation of chemotaxis	9.0E-03	89.76	1	2
	nitrate reductase activity	2.0E-09	193.88	4	5
	nitrate assimilation	2.8E-08	121.18	4	8
	nitrate reductase complex	9.8E-08	242.35	3	3
	nitrate metabolic process	2.3E-05	242.35	2	2
	succinate-CoA ligase (ADP-forming) activity	2.3E-05	242.35	2	2
	protein transport	7.7E-05	40.39	3	18
c	nitrate transmembrane transporter activity	2.2E-04	96.94	2	5
	tricarboxylic acid cycle	4.5E-03	23.08	2	21
	poly-beta-1,6-N-acetyl-D-glucosamine transmembrane transporter activity	4.9E-03	242.35	1	1
	nitrite transmembrane transporter activity	4.9E-03	242.35	1	1
	chaperone-mediated protein complex assembly	4.9E-03	242.35	1	1
	siderophore transmembrane transporter activity	4.9E-03	242.35	1	1
	energy transducer activity	4.9E-03	242.35	1	1
	bacterial-type flagellum assembly	2.8E-10	126.22	5	12
	bacterial-type flagellum-dependent cell motility	1.5E-09	94.67	5	16
	bacterial-type flagellum basal body	5.2E-06	90.88	3	10
	bacterial-type flagellum hook	8.1E-05	151.47	2	4
	structural molecule activity	2.0E-04	100.98	2	6
	cytoskeletal motor activity	3.7E-04	75.73	2	8
d	outer membrane-bounded periplasmic space	1.0E-03	46.61	2	13
	extracellular region	1.6E-03	37.87	2	16
	bacterial-type flagellum basal body, distal rod, L ring	3.8E-03	302.94	1	1
	bacterial-type flagellum basal body, distal rod	3.8E-03	302.94	1	1
	amidase activity	3.8E-03	302.94	1	1
	bacterial-type flagellum basal body, distal rod, P ring	3.8E-03	302.94	1	1
	bacterial-type flagellum filament cap	3.8E-03	302.94	1	1
	viral capsid assembly	6.5E-07	78.18	3	3
	siderophore biosynthetic process	7.9E-06	58.63	3	4
	acid-amino acid ligase activity	1.0E-03	39.09	2	4
	response to stimulus	1.2E-03	26.06	2	6
e	siderophore uptake transmembrane transporter activity	5.4E-03	22.34	2	7
	signaling receptor activity	5.4E-03	22.34	2	7
	ABC-type transporter activity	7.4E-03	15.64	2	10
	cell outer membrane	7.9E-03	4.89	4	64
	polyamine biosynthetic process	1.0E-02	78.18	1	1
	carboxy-lyase activity	1.0E-02	78.18	1	1
	bacterial-type flagellum basal body	0.0E+00	94.25	7	10
	bacterial-type flagellum-dependent cell motility	0.0E+00	84.15	10	16
	cytoskeletal motor activity	2.8E-12	100.98	6	8
	bacterial-type flagellum organization	9.7E-08	76.94	4	7
	bacterial-type flagellum assembly	2.6E-06	44.88	4	12
f	siderophore uptake transmembrane transporter activity	1.0E-04	57.70	3	7
	signaling receptor activity	1.0E-04	57.70	3	7
	bacterial-type flagellum basal body, rod	1.3E-04	89.76	2	3
	chemotaxis	4.0E-04	16.32	4	33
	protein secretion	1.6E-03	17.56	3	23
	plasma membrane	3.5E-03	3.23	10	417

D

#	GO term	P value	Fold enrichment	DEG number in category	All genes in category
	purine nucleobase metabolic process	9.4E-05	82.85	2	3
	L-phenylalanine catabolic process	2.1E-04	82.85	2	3
	metal ion binding	8.3E-04	3.85	8	258
	allantoin metabolic process	4.8E-03	124.28	1	1
	urate catabolic process	4.8E-03	124.28	1	1
	2-oxo-4-hydroxy-4-carboxy-5-ureidoimidazoline decarboxylase activity	4.8E-03	124.28	1	1
	phenylalanine 4-monooxygenase activity	7.4E-03	124.28	1	1
	ribokinase activity	7.5E-03	124.28	1	1
	D-ribose catabolic process	7.5E-03	124.28	1	1
	1-phosphofructokinase activity	7.5E-03	124.28	1	1
	xylan catabolic process	7.7E-03	124.28	1	1
	allantoin catabolic process	7.9E-03	124.28	1	1
g	allantoicase activity	7.9E-03	124.28	1	1
	D-xylose transmembrane transport	8.0E-03	124.28	1	1
	monosaccharide binding	8.0E-03	124.28	1	1
	ABC-type polyamine transporter activity	8.4E-03	124.28	1	1
	citrate synthase activity	8.7E-03	124.28	1	1
	fumarylacetoacetase activity	9.1E-03	124.28	1	1
	beta-alanine-pyruvate transaminase activity	9.2E-03	124.28	1	1
	catalase activity	9.3E-03	124.28	1	1
	hydrogen peroxide catabolic process	9.3E-03	124.28	1	1
	phosphogluconate dehydratase activity	9.8E-03	124.28	1	1
	Entner-Doudoroff pathway through 6-phosphogluconate	9.8E-03	124.28	1	1
	D-gluconate catabolic process	9.8E-03	124.28	1	1
	gamma-aminobutyric acid metabolic process	3.5E-03	193.88	1	1
	4-aminobutyrate:2-oxoglutarate transaminase activity	3.5E-03	193.88	1	1
	ornithine-oxo-acid transaminase activity	3.5E-03	193.88	1	1
h	ornithine(lysine) transaminase activity	3.5E-03	193.88	1	1
	ornithine cyclodeaminase activity	7.2E-03	96.94	1	2
	fatty-acyl-CoA binding	8.1E-03	193.88	1	1
	pyridoxamine-phosphate oxidase activity	9.5E-03	96.94	1	2
i	integral component of membrane	1.4E-05	1.71	42	1164
	oxidoreductase activity	8.0E-03	2.97	7	112
	nitrite reductase (NO-forming) activity	3.9E-04	969.40	1	1
	protein histidine phosphatase activity	7.5E-04	969.40	1	1
j	nitrogen compound metabolic process	5.1E-03	96.94	1	10
	gluconeogenesis	7.0E-03	74.57	1	13
	copper ion binding	7.6E-03	64.63	1	15
	lipopolysaccharide biosynthetic process	1.3E-04	11.10	4	6
	acylphosphatase activity	1.6E-03	16.66	2	2
	cellular biosynthetic process	2.5E-03	9.99	3	5
	4-hydroxybutyrate dehydrogenase activity	3.4E-03	16.66	2	2
k	polysaccharide transport	3.8E-03	16.66	2	2
	tRNA aminoacylation for protein translation	4.2E-03	16.66	2	2
	extracellular space	4.4E-03	16.66	2	2
	quorum sensing	5.7E-03	11.10	2	3
	organonitrogen compound biosynthetic process	6.3E-03	11.10	4	6
	cyclopropane-fatty-acyl-phospholipid synthase activity	9.2E-03	11.10	2	3
	calcium ion binding	1.4E-03	48.96	1	11
l	extracellular region	5.6E-03	33.66	1	16
	toxin activity	5.9E-03	179.52	1	3

Fig. S3 An overview of transcriptome analysis of Δ*fur1*, Δ*fur2* and Δ*fur1*Δ*fur2* under the 450 nM Fe^{2+} conditions.

(A and B) Venn diagrams showing the numbers of differentially expressed genes (DEGs) with the expression level $\log_2$[Δ*fur1*/OE1-1] > 2, $\log_2$[Δ*fur2*/OE1-1] > 2 and/or $\log_2$[Δ*fur1*Δ*fur2*/OE1-1] > 2 with FDR < 0.05 (A), $\log_2$[Δ*fur1*/OE1-1] < -2, $\log_2$[Δ*fur2*/OE1-1] < -2 and/or $\log_2$[Δ*fur1*Δ*fur2*/OE1-1] < -2 with FDR < 0.05 (B). The lowercase characters indicate the gene sets in C and D. (C and D) Enriched Gene Ontology (GO) terms in the up-regulated genes (C) and down-regulated genes (D) with indicated characters in A or B having *P* value < 10^{-3} are listed.
